# Portable X-ray fluorescence (pXRF) calibration for analysis of nutrient concentrations and trace element contaminants in fertilisers

**DOI:** 10.1371/journal.pone.0262460

**Published:** 2022-01-11

**Authors:** Gifty E. Acquah, Javier Hernandez-Allica, Cathy L. Thomas, Sarah J. Dunham, Erick K. Towett, Lee B. Drake, Keith D. Shepherd, Steve P. McGrath, Stephan M. Haefele

**Affiliations:** 1 Department of Sustainable Agriculture Sciences, Rothamsted Research, Harpenden, Hertfordshire, United Kingdom; 2 World Agroforestry (ICRAF), Nairobi, Kenya; 3 Department of Anthropology, University of New Mexico, Albuquerque, NM, United States of America; Soil and Water Resources Institute ELGO-DIMITRA, GREECE

## Abstract

With the increasing popularity of local blending of fertilisers, the fertiliser industry faces issues regarding quality control and fertiliser adulteration. Another problem is the contamination of fertilisers with trace elements that have been shown to subsequently accumulate in the soil and be taken up by plants, posing a danger to the environment and human health. Conventional characterisation methods necessary to ensure the quality of fertilisers and to comply with local regulations are costly, time consuming and sometimes not even accessible. Alternatively, using a wide range of unamended and intentionally amended fertilisers this study developed empirical calibrations for a portable handheld X-ray fluorescence (pXRF) spectrometer, determined the reliability for estimating the macro and micro nutrients and evaluated the use of the pXRF for the high-throughput detection of trace element contaminants in fertilisers. The models developed using pXRF for Mg, P, S, K, Ca, Mn, Fe, Zn and Mo had R^2^ values greater or equal to 0.97. These models also performed well on validation, with R^2^ values greater or equal to 0.97 (except for Fe, R^2^_val_ = 0.55) and slope values ranging from 0.81 to 1.44. A second set of models were developed with a focus on trace elements in amended fertilisers. The R^2^ values of calibration for Co, Ni, As, Se, Cd and Pb were greater than or equal to 0.80. At concentrations up to 1000 mg kg^-1^, good validation statistics were also obtained; R^2^ values ranged from 0.97–0.99, except in one instance. The regression coefficients of the validation also had good prediction in the range of 0–100 mg kg^-1^ (R^2^ values were from 0.78–0.99), but not as well at lower concentrations up to 20 mg kg^-1^ (R^2^ values ranged from 0.10–0.99), especially for Cd. This study has demonstrated that pXRF can measure several major (P, Ca) and micro (Mn, Fe, Cu) nutrients, as well as trace elements and potential contaminants (Cr, Ni, As) in fertilisers with high accuracy and precision. The results obtained in this study is good, especially considering that loose powders were scanned for a maximum of 90 seconds without the use of a vacuum pump.

## Introduction

Fertilisers are an important input used in modern agriculture worldwide. According to the FAO, the Fertiliser Outlook Expert Group forecasts that the world will need 201 million tonnes of N, P and K for fertiliser use in 2022 [1]. Straight fertilisers that contain only one nutrient (for example urea, triple superphosphate or potassium chloride), binary fertilisers containing two macro nutrients (for example diammonium phosphate), compound fertilisers (mostly containing NPK in variable concentrations but each granule contains all elements), and blended fertilisers are commonly used [2]. The latter are mixed from straight, binary or compound fertilisers and other components, to produce for example crop, soil or region specific NPK mixtures. Micronutrients can also be added during the blending process, the most important being molybdenum, zinc, copper, boron and cobalt. However, getting nutrient concentrations in the blending process right requires proper equipment, process management and quality control. Particularly in developing countries, these conditions are not always met. Small blending businesses might not have the resources to buy the right machines, manage the process well enough and/or conduct the necessary quality control. In addition, the fraudulent practice of fertiliser adulteration involves adding extraneous material to a standard fertiliser to lower its nutrient concentration [2]. This is common in Africa and Asia even with commercial blends, which get re-packaged by stockists and sold in smaller quantities. Consequently, considerable problems with local blends have been reported from many countries, resulting in the marketing of fertilisers of inferior quality [3–5]. The sales of substandard fertiliser products reduce farmers’ income, undermine their trust in fertilisers and might even slow down technology adoption [5].

Fertiliser contamination with trace elements is another problem. Trace elements are usually found at low levels in phosphate rock and other raw ingredients of fertilisers. But phosphate rock can contain hazardous metals such as arsenic, cadmium, lead, mercury, chromium and uranium. The concentrations of these metals vary based on the geographical location of the deposit [6]. Trace elements also find their way into fertilisers through the recycling of industrial wastes used for the blending of fertilisers. This can be intended in order to add micronutrients to the fertiliser blend or can be a fraudulent practice to reduce the nutrient concentration of the fertiliser. Depending on the concentration, the frequency of application and retention in the soil, the contaminants can accumulate in the soil and are potentially also taken up by plants. Thus, they can constitute a danger to the environment and to human health. For example, Cd occurs naturally in phosphate rock fertilisers at concentrations ranging between 1–200 mg Cd kg^-1^ P_2_O_5_ [7]. Studies at Rothamsted Research showed that long-term use of superphosphate fertilisers in the Park Grass experiment increased the Cd content of soil and herbage compared with control conditions, and that cadmium can remain plant available for up to one hundred years [8, 9]. Therefore, many countries including the USA, Japan, China, Australia and several European countries have regulations which limit the amount of selected non-nutritive elements in fertilisers, particularly As, Cd, Co, Cr, Pb and Hg [10]. Since 2019, the EU also has requirements to measure trace element contents in fertilisers and conform to limits [11].

The analysis and monitoring of the primary and secondary nutrients, as well as trace elements is therefore necessary to ensure the quality of fertilisers and to comply with local regulations. Conventional wet chemistry methods for this purpose, such as inductively-coupled plasma-optical emission spectrometry (ICP-OES) procedures are available but costly, time consuming and reliable laboratories might not be available locally. Alternatively, we propose to use a rapid, non-destructive portable handheld x-ray fluorescence (pXRF) spectrometer to determine the total elemental composition of fertilisers.

X-ray fluorescence (XRF) analysis of materials with a wide range of elemental contents can give comparable results to those obtained by conventional techniques in inorganic materials [12–17], as well as organic materials [18, 19] and fertilisers [10, 20–22]. Additionally, pXRF instruments are now available and can be used in-situ for quality control and environmental assessments, yet still have performance levels approaching those of benchtop devices [16, 17]. In-situ environmental applications include the analysis of contaminated soils/sediments/waste materials [12, 13, 15–17, 23–25]. This in-situ technique could especially benefit developing countries where market and environmental monitoring is often limited by a scarcity of specialized laboratory facilities and field scientists [23].

The validity of non-destructive XRF analysis to the part-per-million (ppm) level has been essential in geochemical provenance studies of stone tools [26, 27]. However, there are few reports of the use of these devices in agronomy, and they are primarily constrained to ‘proof-of-concept’ studies. But tests of its applicability have demonstrated that it can evaluate low-ppm levels of toxic metals [28, 29]. In assessing trace elements in certified reference materials (CRM) soils it was observed that pXRF was equivalent to or better than ICP-OES in measuring Ti, Cr, Mn, Fe, Cu, Zn, Sr, Cd, and Pb, and detection limits ranged from 2 to 10 ppm in Cd and Ni, respectively [17]. Therefore, pXRF spectroscopy could enable quick and cheap determination of macro- and micronutrient composition of fertilisers, and at the same time identify possible contamination with trace elements. Consequently, the objectives of our study were to a) develop empirical calibrations for a pXRF tool using a large range of standard fertilisers (“Fertiliser model”) and amended fertilisers (“Fertiliser Trace model”), b) determine the reliability of the measurements for macro and micro nutrient elements from Na to Mo using a validation set and c) evaluate the use of the pXRF for the detection of trace element contaminants in fertilisers.

## Materials and methods

### Overview of the fertiliser materials

Multi-element fertilisers encompassing a wide range of atomic weights and densities were used as reference materials for the development of Lucas-Tooth empirical quantification models [30] following protocols outlined in [31]. A list of the fertilisers is provided in Table 1. The densities of the fertilisers ranged from 0.86 g cm^-3^ for EDTA to 3.71 g cm^-3^ for Cobalt (II) sulphate, Table 1. Density affects the depth of penetration for emitted x-rays returning from the sample; as such, XRF signal count rates vary significantly among fertiliser types. This makes fertilisers one of the most challenging matrices to analyse with XRF.

**Table 1:** Overview of fertilisers used for pXRF models calibration and validation.

Fertiliser	Major elements	Bulk density (kg m^-3^)	Density (g cm^-3^)
Mavuno planting^&^	P, K, Ca	-	-
Peri urban leafy vegetables^&^	K, P, Ca, S	-	-
Organic fertiliser^&^	Ca, K, Fe	-	-
Calcium ammonium nitrate	Ca. N*	1000–1100	1.71
Sulphate of ammonia	S. N*	785–1100	1.77
Triple superphosphate	P, Ca, Mg, Fe, S	950–1200	-
Muriate of potash	K, Na	1030–1345	1.98
Diammonium phosphate	P, S, Ca. N*	880–1100	1.62
NPK	K, P, Ca, S. N*	-	-
Mijingu composite fertiliser^&^	Ca, P, Mg, S, K	-	1.02–1.14
Boric acid	B	860–1010	1.44
Calcium chloride hexahydrate	Ca	960	1.71
Di-sodium hydrogen orthophosphate anhydrous	Na, P	880	1.70
Ethylenediaminetetraacetic acid (EDTA) ^&^	Fe, Na	800–1000	0.86
Magnesium sulphate	S, Mg	830–1300	1.50–2.66
Potassium sulphate	K, S	1440	2.66
Sodium chloride	Na. Cl*	1280–1300	2.17
Borax	Na, B	960	1.73–2.40
MOP	K, Na	1030–1200	2.00
ICR176581^&^	Ca, P, Mg, S	-	-
ICR176582^&^	K, Ca, P	-	-
ICR176583^&^	P, S	-	-
ICR176584^&^	P, S	-	-
Urea^&^	K, Ca, P, S. N*	720–820	1.32
SOP	K, S	1270	2.66
Ammonium molybdate tetrahydrate	Mo, K. N*	1400–1600	2.50
Cobalt (II) sulphate	Co, S, K	770	3.71
Copper (II) sulphate	Cu, S	830	3.60
Manganese (II) sulphate hydrated	Mn, S	1120	2.17
Zinc sulphate heptahydrate	Zn, S, Na	1330	1.97–2.07
ZnSo4	Zn, S, Fe, Na	1330	3.54
Double top	S. N*	785–1100	-
Polysulphate	S, Ca, K, Mg, Na	1599	-
Kieserite	S, Mg, Na, K	1110	2.57
DAP	P, S, Ca. N*	880–1100	1.62
Nitram	N*	900–1000	1.72
MOP	K	1030–1200	2.00
TSP	P, Ca, S	950–1200	2.20
TSP RRes	P, Ca, S	950–1200	2.20
Limestone grit	Ca	960	1.50–2.70
Ammonium sulphate RRes	S. N*	785–1100	1.77
Nitrochalk	Ca, Mg, S. N*	1000–1100	-
Potassium sulphate RRes	K, S	1440	2.66
Zinc sulphate	Zn, S	1330	3.54
Ammonium sulphate	S. N*	785–1100	1.77
Urea^&^	N*	720–820	1.32
Sodium chloride	Na. Cl*	1280–1300	2.16
Boric Acid	None**	860–1010	1.44
Manganese (II) sulphate hydrated	Mn, S	1120	3.25
Magnesium sulphate	S, Mg	830–1300	2.66
Ammonium molybdate	Mo. N*	1400–1600	2.50
Calcium chloride hexahydrate	Ca. Cl*	960	1.71
Sovereign sulphur S1	S	1100–1300	2.07
Sovereign sulphur S2	S	1100–1300	2.07
Brimstone 90	S	1120	2.07
SulFer 95	S	1100–1500	2.07

Elements with concentration ≥ 10,000 mg kg^-1^ (measured by ICP-OES) are reported in decreasing order. Density and bulk density are nominal, as reported in the literature.

* Denotes major elements expected, but not measured in this study.

** This boric acid had K as its main nutrient, with a concentration of 201 mg kg^-1^.

^&^ Denotes carbon containing fertilisers.

Of the 56 fertiliser samples used, 31 were sourced by the World Agroforestry (ICRAF), Nairobi, Kenya and the remaining 25 samples were sourced by Rothamsted Research (RRes), Harpenden, UK. Samples for calibration and validation were selected considering the different fertiliser types (e.g. sulphates, phosphates, nitrates, etc). Thirty-nine fertilisers were used for calibrating and 17 for validating a model that will be referred to as “Fertiliser”. Ten of the fertilisers to be used for model calibration were amended with 6 elements of interest to cover a wider range of concentrations, related details are given in section 2.3. This gave an additional set of 187 samples for the calibration (n = 100) and validation (n = 87) of a second model with a focus on concentrations from 0–1000 mg kg^-1^ of Se and Co, as well as Ni, As, Cd and Pb (i.e. potential contaminants). This second model will be called “Fertiliser Trace”.

### Aqua Regia digestion and ICP-OES

Fertiliser samples for reference were ground to pass a 75-microns mesh sieve on a Retsch PM400 planetary ball mill (Retsch GmbH, Germany). Dry powdered test samples (0.1000 ± 0.0005 g) were digested in graduated Pyrex tubes (150 x 20 mm) with 5 ml of high purity Aqua Regia acid mixture [32]. First, 4 ml of concentrated HCl (s.g. 1.18, 37%) was added to each tube tilting the tube to maximize contact with the powdered sample. Then, 1 ml of concentrated HNO_3_ (s.g. 1.42, 70%) was added to each tube and manually mixed with a gentle handshaking. The tubes were placed in a Carbolite heating block with Eurotherm 818 Controller/Programmer allowing slow ramp rates and a final temperature of 120°C to complete the digestion. After cooling, 5 ml of 20% (v/v) HNO_3_ was added, the tube was mixed and rewarmed at 80°C and ultra-pure water (18 MΩ H_2_O) was added to give 25 ml of a clear solution in a final 5% (v/v) HNO_3_ matrix. Sample replicates were included every 10 samples. Each set of samples also included in duplicate a digestion blank (only digestion solution in the tube) and an internal reference sample (in house WO3 HRM1) for quality and possible contamination check. Total concentrations of Na, Mg, Al, P, S, K, Ca, Ti, Cr, Mn, Fe, Co, Ni, Cu, Zn, As, Se, Mo, Cd and Pb in digested solutions were then determined by ICP-OES (ICP-OES, Perkin Elmer Optima 7500 DV, Waltham, MA, USA). The analytical procedures gave satisfactory recoveries for all the included reference materials (within ± 2 times standard deviation of the mean value for each reference element). Descriptive statistics of the elements of interest as determined by ICP-OES are presented in Table 2. These original fertilisers were used in the calibration (39 samples) and validation (17 samples) of the first pXRF model called “Fertiliser”.

**Table 2:** Descriptive statistics of elements of interest in the fertiliser calibration model.

	Calibration set (n = 39)			Validation set (n = 17)		
Element	Mean	SD	Range	Mean	SD	Range
Na (%)	3.8	9.8	0–40.2	2.5	9.5	0–39.1
Mg (%)	1.9	4.2	0–15.8	1.2	3.4	0–14
Al (%)	0.2	0.3	0–0.9	0.2	0.3	0–0.8
P (%)	7.6	8.6	0–22.4	2.4	6.6	0–20.3
S (%)	12.3	20.9	0–87.7	17.4	27.5	0–88
K (%)	9.9	17.3	0–51.7	3.3	10.6	0–42.8
Ca (%)	7.1	10.4	0–41.7	3.1	6.4	0–18.5
Ti (mg kg^-1^)	118	173	0.3–572	41	106	0.3–439
Cr (mg kg^-1^)	61	101	0–357	37	85	1.4–293
Mn (%)	1.5	7.2	0–35.2	1.7	7.1	0–29.4
Fe (%)	1.0	3.2	0–15.7	0.1	0.2	0–0.7
Co (%)	3.1	10.7	0–37.1	0.0	0.0	0
Ni (mg kg^-1^)	14	21	0–63	8	13	0.3–49
Cu (%)	1.8	8.5	0–40.9	0.0	0.0	0
Zn (%)	2.8	8.9	0–32.2	1.5	6.2	0–25.7
As (mg kg^-1^)	14	17	0–44	18	56	0.4–232
Se (mg kg^-1^)	272	463	0–1052	15	43	0.2–174
Mo (%)	1.3	5.9	0–27	2.0	8.0	0–33.1
Cd (mg kg^-1^)	6	9	0–28	3	7	0–25
Pb (mg kg^-1^)	28	83	0–291	30	111	0–459

### Amending fertilisers, microwave digestion and ICP–OES/MS

Ten of the fertilisers to be used for model calibration were amended with 6 elements of interest (Co, Ni, As, Se, Cd and Pb) to give a good distribution of concentrations up to 1000 mg kg^-1^. The amended fertilisers were SOP, TSP, Kieserite, Nitram, DAP, Polysulphate, MOP, Nitrochalk, ammonium sulphate, and also limestone. For this purpose, 40 g of each of the fine powdered fertilisers were amended with 1090 mg of a cocktail of chemical salts (between 80 to 250 mg of each salt depending on the target element and salt) to give target concentrations of approximately 10 mg kg^-1^, 50 mg kg^-1^, 100 mg kg^-1^, 200 mg kg^-1^, 400 mg kg^-1^, 600 mg kg^-1^ and 1000 mg kg^-1^. Table 3 presents the chemical salts used and the actual concentrations achieved in the amended samples for each element. The amended samples were thoroughly mixed in plastic bags, separated in sets of 5 g and milled on a Retsch PM400 ball mill 3 times for 6 min at 250 rpm. Between each of the 3 milling events, the 5 g subsamples were bulked and thoroughly mixed again in a plastic bag. This process was repeated in duplicate to check reproducibility. Different quantities of the first set of amended samples were thoroughly mixed with different quantities of corresponding non-amended original fine ground powdered fertiliser to obtain 10 g mixtures with different concentrations of the target elements. Each mixture was thoroughly mixed in plastic bags, separated in sets of 5 g and milled on a Retsch PM400 planetary ball mill 3 times for 6 min at 250 rpm. Between each of the 3 milling events all the 5 g subsamples were bulked and thoroughly mixed again in a plastic bag.

**Table 3:** Chemical salts used for spiking and actual concentrations in the amended fertilisers for each element.

Element	Salt used	Elemental concentrations (mg kg^-1^) in the final standards mixtures								
Cd	3CdSO_4_ x 8H_2_O	0.00	11.0	54.8	109.5	219.1	438.1	657.2	876.3	1095.3
Ni	NiSO_4_ x 6H_2_O	0.00	10.1	50.2	100.5	201.0	401.9	602.9	803.9	1004.8
Co	CoSO_4_ x 7H_2_O	0.00	10.5	52.4	104.8	209.7	419.3	629.0	838.6	1048.3
Pb	(CH_3_COO)_2_ Pb_3_H_2_O	0.00	10.9	54.6	109.3	218.5	437.0	655.5	874.0	1092.5
As	Na_2_HAsO_4_ x 7H_2_O	0.00	10.8	54.0	108.1	216.1	432.2	648.3	864.5	1080.6
Se	Na_2_SeO_4_	0.00	10.5	52.3	104.5	209.0	418.0	627.0	835.9	1044.9

In addition, the 1000 mg kg^-1^ samples of 8 out of the 10 amended fertilisers were further diluted with appropriate quantities of the original fertilisers to give target concentrations of 2.5 mg kg^-1^, 5 mg kg^-1^, 7.5 mg kg^-1^, 12.5 mg kg^-1^, 15 mg kg^-1^, 20 mg kg^-1^, 30 mg kg^-1^, 40 mg kg^-1^, 60 mg kg^-1^, 70 mg kg^-1^, 80 mg kg^-1^ and 90 mg kg^-1^. On few occasions when material was limited, the 100 mg kg^-1^ sample of an amended fertiliser was diluted instead. For the amended/diluted fertilisers, test sets of 0.2500 ± 0.0005 g were acid digested in Teflon microwave tubes with a MARS6 CEM microwave equipment (CEM Microwave Technology Ltd., UK). Each digestion tube received 3 ml of concentrated HNO_3_ (SG 1.42, 70%), 2 ml of ultrapure hydrogen peroxide (30% w/v, 100 volumes) and 2 ml of ultra-pure water (18 MΩ H_2_O). The microwave programme was set up to 65 min with a maximum temperature of 140°C that was reached at slowly increasing steps, first to 55°C and then to 140°C. After cooling, completely digested samples were transferred to 50 ml graduated Greiner tubes (Sigma-Aldrich, UK) and ultra-pure water (18 MΩ H2O) was added to give 25 ml of a final clear solution which was used for ICP-OES and ICP-mass spectroscopy analysis (ICP-MS, Perkin Elmer NexION 300X, Waltham, MA, USA).

Descriptive statistics of the elements of interest as determined by ICP-OES/MS are presented in Table 4. These amended/diluted fertilisers were used in the calibration (100 samples) and validation (87 samples) of a second pXRF model called “Fertiliser Trace”.

**Table 4:** Descriptive statistics of elements of interest in the Fertiliser Trace model.

Element	Calibration set (n = 100)			Validation set (n = 87)		
	Mean	SD	Range	Mean	SD	Range
Mg (%)	8.7	7.2	1.9–16.4	4.4	0.1	4.3–4.5
P (%)	20.0	0.4	19.3–20.7	19.8	0.5	19.0–20.6
S (%)	11.2	10.1	1.1–21.6	19.5	1.6	17.7–21.5
K (%)	26.3	24.5	0.1–50.5	27	15.4	11.8–42.7
Ca (%)	21.4	17.9	4.6–40.5	6.8	6.9	0–14.2
Cr (mg kg^-1^)	58	98	0.6–244	73	127	0.7–296
Mn (mg kg^-1^)	9	9	46–388	6	8	0.1–20
Fe (mg kg^-1^)	559	723	71–1892	729	1198	7–2864
Co (mg kg^-1^)	225	316	0.3–1077	189	308	0.3–1119
Ni (mg kg^-1^)	221	301	0.6–1035	189	291	0.2–1069
Cu (mg kg^-1^)	10	17	0.1–44	14	24	0–55
Zn (mg kg^-1^)	85	131	0.4–329	107	187	0.2–435
As (mg kg^-1^)	244	340	0.4–1159	195	310	0.3–1165
Se (mg kg^-1^)	256	377	1–1943	180	294	0–1171
Mo (mg kg^-1^)	2	2	0.1–6	9	2	0–4
Cd (mg kg^-1^)	275	380	0.2–1278	223	358	0.2–1361
Pb (mg kg^-1^)	240	338	0–1166	192	316	0–1164

### pXRF data collection

Portable XRF analysis was conducted using the Bruker Tracer 5i (Bruker Corp., USA) handheld instrument equipped with a 2 W Rh tube and a silicon drift detector (SSD). The instrument had a full width height maximum (FWHM) of 135 eV at the Manganese K-alpha line. In most XRF analysis, excitation parameters are optimized for either light (Na—Ca) or heavy (Ti—U) elements [33, 34]. Prior to spectra collection, test samples ground to pass a 75-microns mesh sieve were dried in a conventional oven at 40 ^0^C for 24 hours. All pXRF data were undertaken by placing loose powdered samples in Chemplex XRF cups with Prolene® film at the base, on top of the instrument, which sat in a stand with the beam pointing upwards, under normal laboratory conditions using two different scanning parameters, Table 5, for the two models (i.e. “Fertiliser” and “Fertiliser Trace”). Calibration elements of interest were Na, Mg, Al, P, S, K, Ca, Ti, Cr, Mn, Fe, Co, Ni, Cu, Zn, As, Se, Mo, Cd and Pb. Ti and Al were of interest because they tend to occur as impurities in organic fertilisers or accumulate during the manufacturing of inorganic fertilisers from rocks.

**Table 5:** Scanning parameters used for pXRF data collection.

	Fertiliser	Fertiliser Trace
Voltage (kV)	35	40
Current (μA)	30	26
Time (seconds)	60	90
Filter	None	Ti 25 μm: Al 300 μm
Atmosphere	Air	Air

### Model calibration and validation

Using conventional wet chemistry (reference) data and data obtained with the pXRF, Bruker’s EasyCal software was used to develop empirical Lucas-Tooth quantification models [30] following protocols outlined in [31].

Based on the Lucas-Tooth and Price algorithm, pXRF concentration was computed as:

$$C_{i}=r_{0}+I_{i}\;(r_{i}+\sum r_{r}n\;*\;I_{r}n)+e_{q}+e_{c}+e_{h}+e_{i}$$ Eq 1

where for element *i*, C_i_ is the concentration, r_0_ is the empirical constant (intercept), I_i_ is the net intensity, r_i_ is the empirical coefficient for intensity, r_r_n is the empirical constant for effect of element n on element i, and I_r_n is the net intensity of element n. The four error terms originate from the quantification procedure (e_q_), counting statistics (e_c_), heterogeneity in the sample (e_h_) and external errors from other sources (e_i_) such as sample orientation to the beam, instrumental quality, etc. For energy-dispersive XRF (ED-XRF), e_c_, which is the noise in count rates near the element in question can be reduced by extending measurement time. e_h_, can be addressed by sample preparation and taking multiple measurements on the sample per the general guide to uncertainty in measurement (GUM). e_h_ is dependent on both the quality of the samples and the application of inter-elemental corrections. The above equation assumes knowledge of the variation of other elements because some elements influence the fluorescence of others. One major advantage of the Lucas-Tooth algorithm is that it corrects for these effects by producing linear models for the quantification of each element; consequently, calibrations will be accurate within the range of the regression line.

EasyCal enabled custom matrix correction, as well as background, Compton and overlap corrections using measured intensities of the reference materials. The Kα lines of the elements of interest were used for calibrations, except for Pb, for which the Lα line was used. The performance of the “Fertiliser” and “Fertiliser Trace” models were validated with independent test-sets comprised of 17 fertilisers and 87 amended/diluted fertilisers respectively. Lastly, the validated models were used to determine the elemental composition of an internationally certified reference material, NIST SRM 695, a multi-nutrient blended fertiliser. The relative standard deviation (RSD) of the sample mean was used to assess the precision of the models. In addition, the % difference (% D) was adopted to classify the adequacy of pXRF in analysing fertilisers [35].

## Results and discussion

### Qualitative analysis

**Fig 1:**
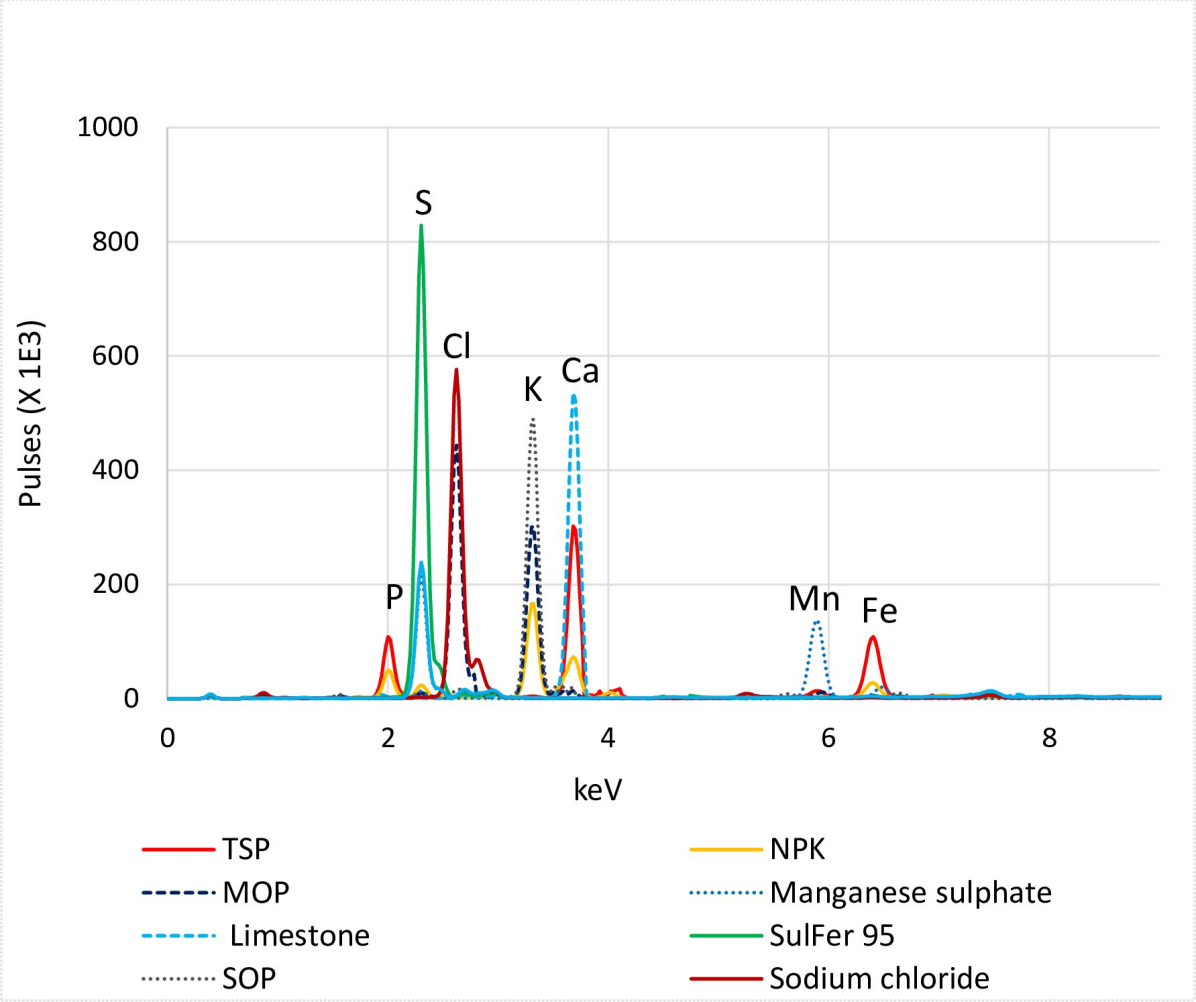
pXRF spectra with energy lines for selected elements. Phosphorus P, sulphur S, chloride Cl, potassium K, calcium Ca, manganese Mn and iron Fe in eight different fertilisers.

Based on the spectra, pXRF can be used as a rapid screening tool to give an indication of the elemental composition of fertilisers. The peak height and area of an energy line provides insight about the relative concentration of the element of interest; as seen for instance for sulphur in sulfer95 versus SOP, and calcium in Limestone, TSP and NPK, Fig 1. With the appropriate scanning parameters, a low power pXRF can detect elements from Na to Pb even if an element is not of interest, as observed in the case of the Cl peak in MOP.

### Quantitative analysis

#### Fertiliser model

Fitting statistics obtained for empirical models developed using pXRF spectra and conventional ICP-OES/MS data are presented in Table 6. The R^2^ and slope of validation (predicted versus observed for the independent sample set) were used to evaluate the performance of the calibration models developed. The R^2^ provides information regarding model precision whereas the slope gives indication of the model’s accuracy. For the “Fertiliser” model, the R^2^ values of calibration for the macro and micronutrients were greater or equal to 0.97 for Mg, P, S, K, Ca, Mn and Fe. These models also performed very well on validation, with R^2^ values greater or equal to 0.97 and slope values ranging from 0.81 to 1.44; except for Fe which had an R^2^ value of 0.55. Poor calibration statistics were however obtained for Na, Al, Ni, Se, Cd and Pb. This could be attributed to the fact that, the “Fertiliser” calibration targeted macro and secondary elements and the fertilisers used did not have a uniform distribution of data points for the micro/trace elements allowing a good calibration; thus a wider range of reference materials are needed to define and refine these curves. Na on the other hand is too light to be reliably detected, Fig 1 or quantified, Table 7, by using the scanning parameters employed in this study. Samples were scanned in air, and the argon in air causes the attenuation of fluorescent energy of low atomic number (Z) elements [36]. To improve sensitivity towards low Z elements, a vacuum pump can be used to evacuate the beam path. The introduction of silicon drift detectors coupled with Rh thin window X-ray tubes promised to enable the detection of Na without having to measure in vacuum, but this was not realised in the current study.

**Table 6:** Goodness of fit parameters for empirical pXRF “Fertiliser” and “Fertiliser Trace” models.

	Fertiliser model				Fertiliser Trace model	
	Calibration		Validation		Calibration	
Element	R^2^_Cal_	SD	R^2^_Val_	Slope	R^2^_Cal_	SD
Na (%)	0.11	0.4	-	-	-	-
Mg (%)	0.97	0.7	0.99	1.23	0.99	0.6
P (%)	0.97	1.2	0.98	1.15	0.95	1.6
S (%)	0.99	0.7	0.99	1.04	0.99	1
K (%)	0.99	1.2	0.99	0.97	0.99	0.9
Ca (%)	0.97	1.3	0.97	1.44	0.99	1
Mn (%)	0.99	0	0.99	0.86	0.94	2
Fe (%) *	0.99	0.1	0.55	0.95	0.9	231
Cu (%) *	0.99	0	< 0.10	0.27	0.97	3
Zn (%) *	0.99	0	0.99	0.81	0.99	9
Co (%) *	0.99	0.1	-	-	0.97	41
Se (mg kg^-1^)	0.1	463	-	-	0.91	109
Mo (%) *	0.99	0.2	0.99	1.21	0.76	0
Al (%)	0.38	0.1	-	-	-	-
Ti (mg kg^-1^)	0.79	100	< 0.10	< 0.10	-	-
Cr (mg kg^-1^)	0.93	24	0.66	0.7	0.99	26
Ni (mg kg^-1^)	0.11	4	-	-	0.88	102
As (mg kg^-1^)	0.92	5	0.15	1.92	0.86	119
Cd (mg kg^-1^)	0.34	0	-	-	0.9	122
Pb (mg kg^-1^)	0.27	86	-	-	0.91	89

* Unit is mg kg^-1^ for Fertiliser Trace.

The Fertiliser models for Na, Al, Ni, Se, Cd and Pb were not validated because the calibration statistics were below satisfactory. For Co no validation statistics is provided because concentrations were too low (< 1 mg kg^-1^) in the validation set to be quantified by the pXRF.

**Table 7:** Elemental composition (%) of fertilisers as determined by pXRF, ICP and as reported for marketing purposes.

Element	Fertiliser	pXRF	ICP-OES	Nominal
P	DAP	18.5	19.7	20.0
	TSP	16.2	20.3	21.0
S	Double top	13.0	11.4	12.0
	Polysulphate	19.9	20.5	19.2
	DAP	0.9	1.2	-*
	Sovereign sulphur S2	81.5	85.5	90.0
K	Polysulphate	14.0	12.0	11.6
	Ammonium sulphate	0.4	0.6	-*
	Potassium sulphate	44.3	42.8	46.1
Ca	TSP	13.0	16.6	15.0
	Polysulphate	8.0	13.9	12.2
	DAP	1.2	1.1	-*
	Calcium chloride hexahydrate	12.2	18.5	14.0
Na	TSP	0.4	0.5	-*
	DAP	0.3	0.4	-*
	Borax	0.1	16.7	15.6
Mg	Magnesium sulphate	12.0	14.0	15.0
	Polysulphate	3.6	4.2	3.6
	TSP	1.2	0.8	-*
Mn	Manganese sulphate	34.4	29.4	32.0
Fe	TSP	0.2	0.2	-*

-* No nominal value reported.

In addition, even though good calibration statistics were also obtained for the trace elements Cr, Co, Cu, Zn and Mo, only Zn and Mo performed well on validation. Thus, the scanning parameters used for the “Fertiliser” model could also not adequately measure most trace elements (Na, Co, Ni, Al, Cu, Se, Cd, Pb), they were just not present in enough samples of the fertiliser validation set or their concentrations were too low (<1 mg kg^-1^).

The elemental compositions of some fertilisers determined by the in-house calibration developed in this study are presented in Table 7, together with ICP reference values and nominal values for comparison. The results show a good agreement between pXRF, ICP-OES and nominal values given by producers; with pXRF over or underestimations of nominal values usually < 10%. The results also show that smaller concentrations of important nutrient elements not even mentioned in the nominal fertiliser analysis can be detected easily.

#### Fertiliser Trace model

**Fig 2:**
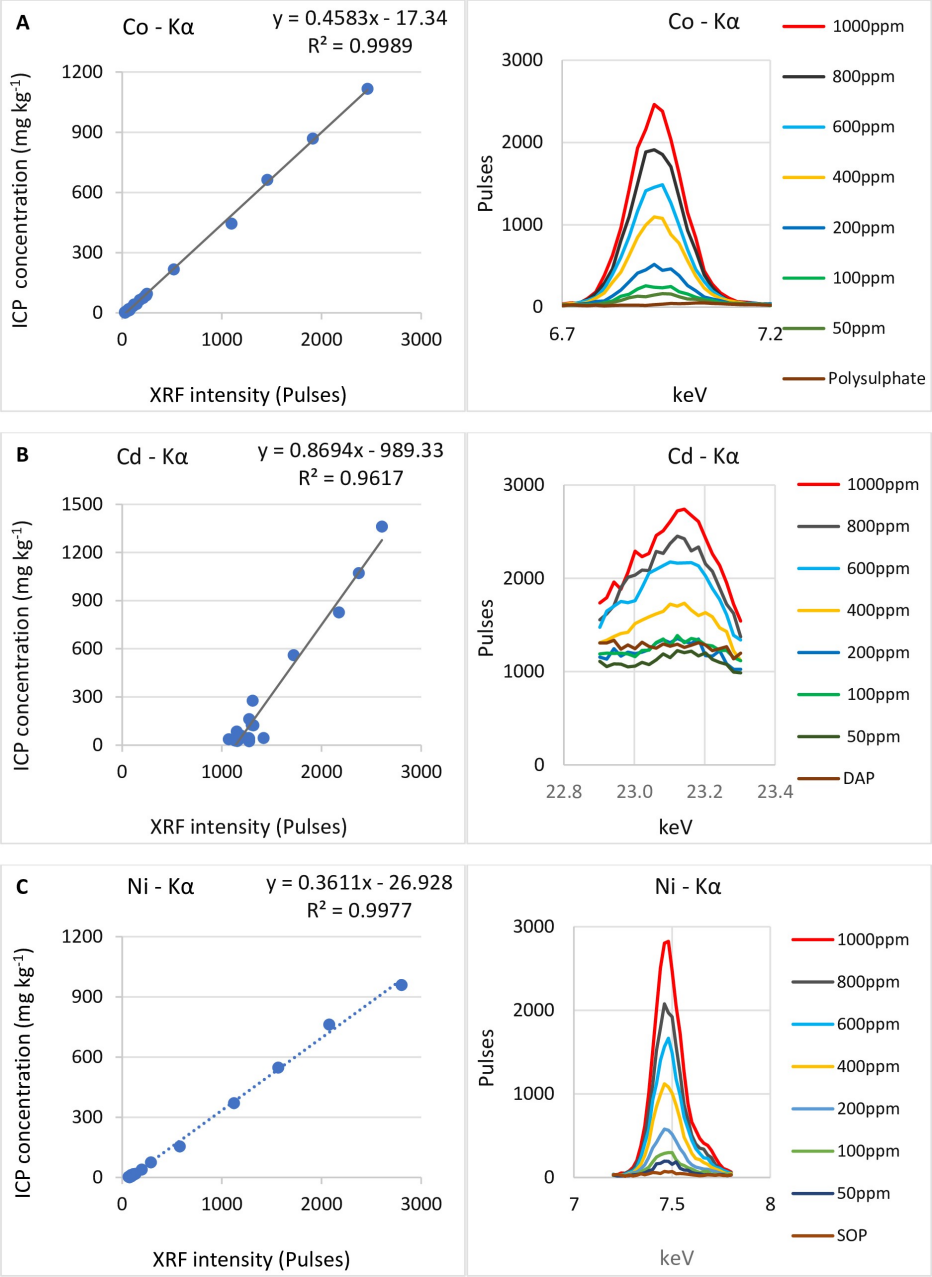
Plots of XRF pulses versus elemental concentrations up to 1000 mg kg^-1^. A–Co in Polysulphate; B–Cd in DAP; C–Ni in SOP.

The “Fertiliser Trace” model was developed by employing scanning parameters known to optimize the detection and measurement of trace elements and contaminants occurring at concentrations up to 1000 mg kg^-1^. For instance, by using a Ti/Al filter, undesired parts of the excitation spectrum were suppressed (via the absorbance of low energy x-rays) for optimum transmittance and detection of the high energy x-rays that are of interest in this case [33]. Fig 2 shows simple regression plots between XRF intensity at the Kα line (i.e. an input of the Lucas-Tooth model) of some elements and ICP reference values. In all cases of the 6 elements of interest (Co, Ni, As, Se, Cd and Pb) in 10 fertilisers (all charts not shown), the calibration of concentrations 0–1000 mg kg^-1^ is highly significant and linear. Symmetric and smooth pulse peaks were observed for all elements except for Cd, which also had a baseline shift. The appearance of the Cd peaks could be explained by the rhodium tube used in this pXRF; the Compton/Raleigh scatter of Rh lines of the x-ray tube is in proximity to the Kα line of Cd [34]. In addition, Pd from some components of the instrument interferes with Cd. This study thus suggests that, the specifications/design of the Tracer 5i (2016 model) limits its application for the analysis of Cd at low concentrations. Notwithstanding, a more aggressive filtration of a spectrum can be achieved by using a black filter (Nb 100 μm, Cu 150 μm, Ti 25 μm and Al 200 μm) which will sharply change the spectrum at the expense of other elements; baseline caused by Rh can be completely eliminated by the black filter.

**Fig 3:**
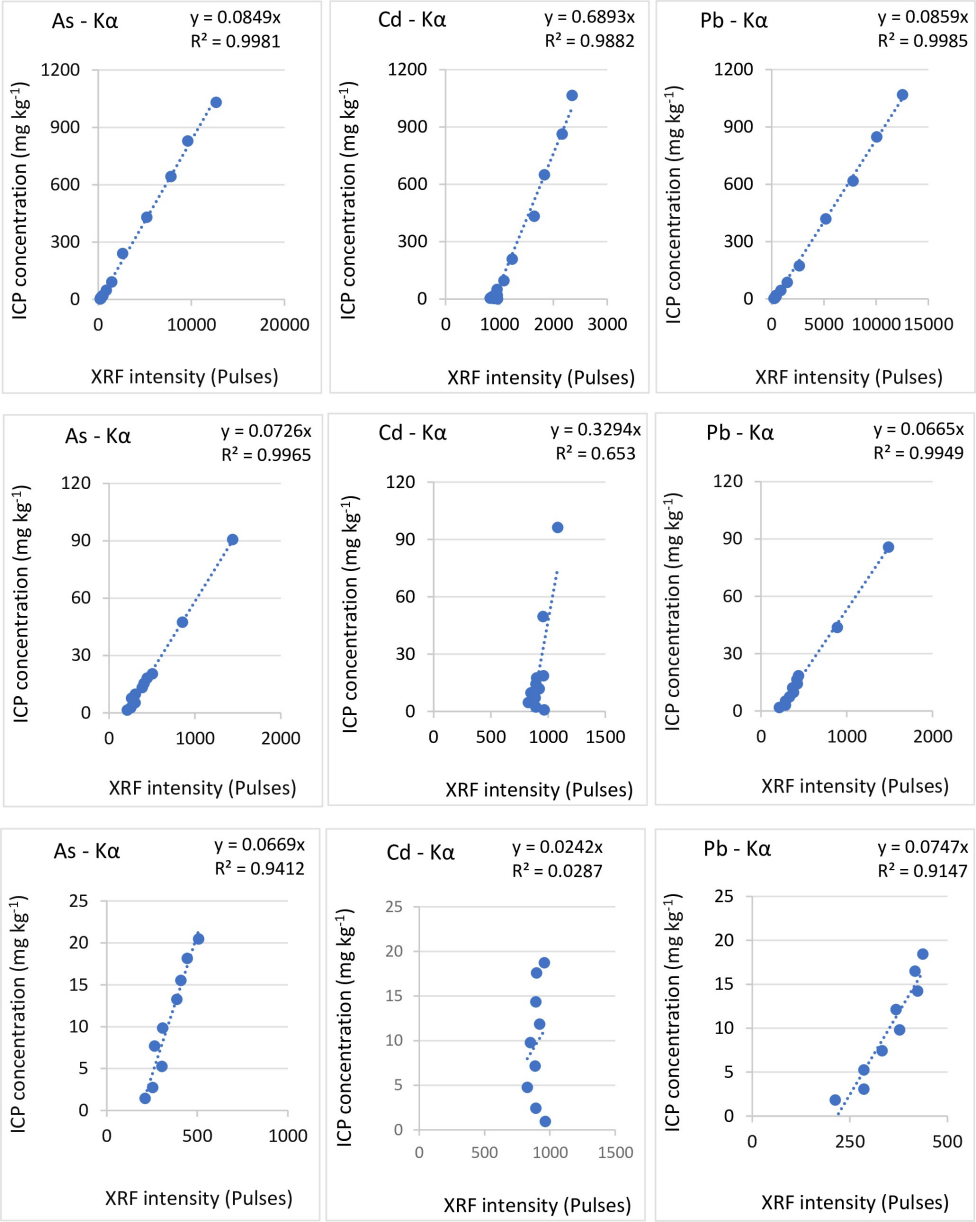
Correlation between XRF intensity and ICP concentration. As, Cd and Pb in SOP for three different concentration ranges; 0–1000 mg kg^-1^ in the top row, 0–100 mg kg^-1^ in the middle row and 0–20 mg kg^-1^ in the bottom row.

The scanning parameters utilized were also able to model reduced concentrations up to 100 mg kg^-1^ and then below 20 mg kg^-1^ for all elements of interest, albeit to a relatively lesser extent except for Cd; examples of which can be seen in Fig 3, showing the correlation plots of XRF pulses and known concentrations of As, Cd, and Pb in amended SOP. Good linear regressions are shown for As and Pb in all three concentration ranges whereas the correlation for Cd is only acceptable at the 0–1000 mg kg^-1^ concentration range.

The R^2^ values of calibration for Co, Ni, As, Se, Cd and Pb models were 0.97, 0.88, 0.80, 0.91, 0.91 and 0.91 respectively, Table 6. In addition, similarly good fitting statistics were obtained for the major and micro elements as well, with R^2^ values ranging between 0.78 to 0.98.

**Fig 4:**
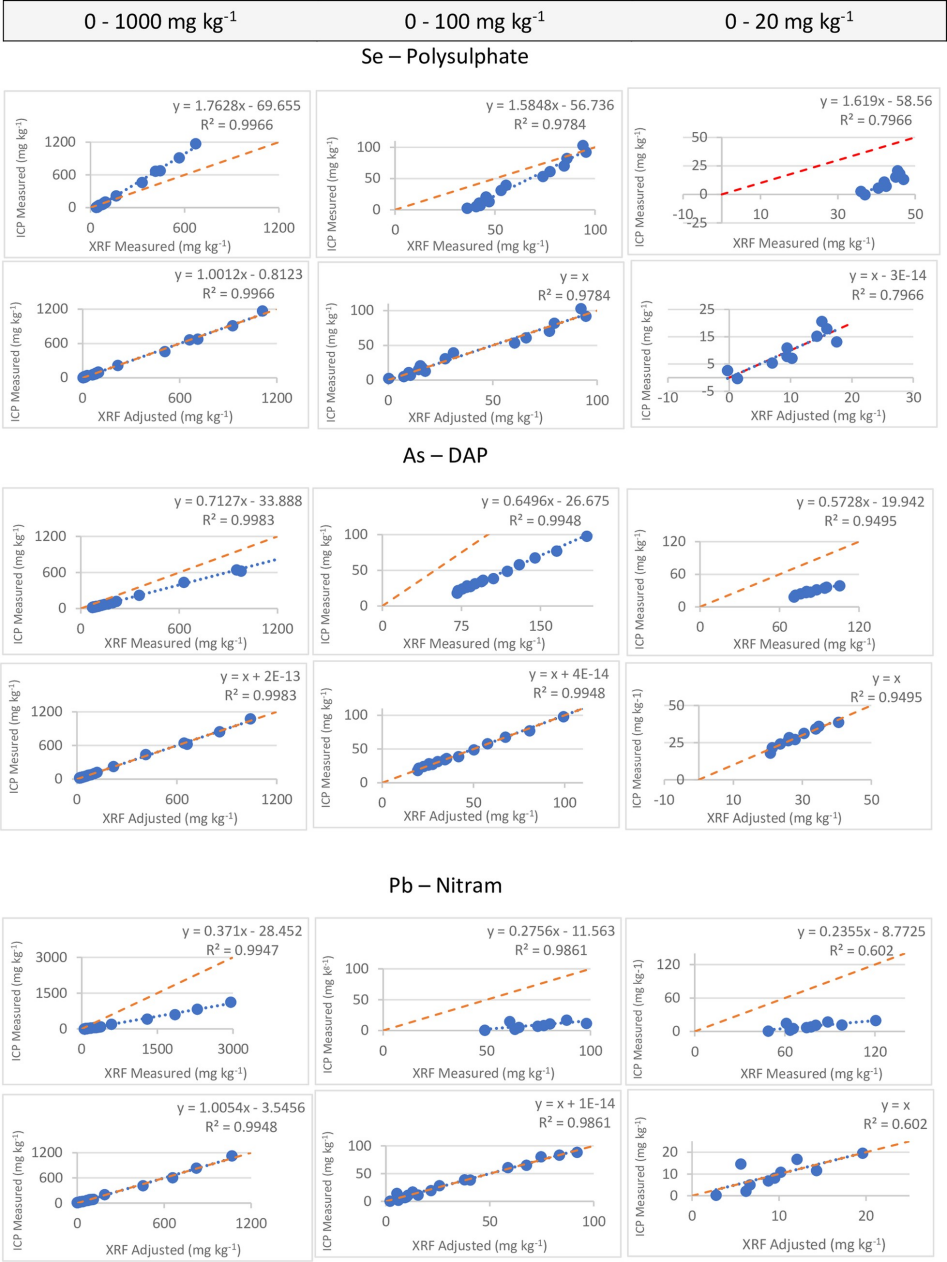
Comparison of some contaminants measured by ICP-MS versus pXRF in fertilisers used for independent validation. Plots also show how slope adjustment improved accuracy. The dashed line indicates the expected 1:1 ratio.

Four amended fertilisers; Polysulphate (S—20%, Ca—14%, K—12%), DAP (P—20%), Nitram (N—35%) and SOP (K—42%, S—18%) were used for independent validation of these models. Regression analyses were conducted for these fertilisers individually. At concentrations up to 1000 mg kg^-1^, R^2^_val_ values for all elements for the 4 fertilisers were greater or equal to 0.98, except for Ni in Nitram which had an R^2^_val_ of 0.52. The slopes of these equations however ranged from 0.37 to 1.97, Table 8. A custom Bruker equation was thus applied to adjust the slopes for this validation set, Table 8. Fig 4 shows a graphic example of how the accuracy of the models were improved with the slope adjustment. The regression coefficients of the validation also had good prediction for the elements of interest in the range of 0–100 mg kg^-1^, with the lowest R^2^ of 0.76 for Cd in SOP. The adjusted slopes had values ranging from 0.99 to 1.14. Cd could not be validated in DAP and Nitram because pXRF gave below detection limit (<LOD) for these fertilisers. At lower concentrations up to 20 mg kg^-1^, the pXRF did not perform as well in measuring contaminants in fertilisers, especially for Cd. Consequently, at such low concentrations, Cd contamination would need to be confirmed by wet chemistry analysis.

**Table 8:** Goodness of fit parameters for the validation of the Fertiliser Trace models.

		0–1000 mg kg^-1^			0–100 mg kg^-1^			0–20 mg kg^-1^		
Element	Fertiliser	R^2^_Val_	Slope	Slope_ADJ_	R^2^_Val_	Slope	Slope_ADJ_	R^2^_Val_	Slope	Slope_ADJ_
Co	Polysulphate	0.99	1.79	1	0.98	1.6	1	0.94	1.69	1.04
	DAP	0.99	0.77	1	0.99	0.71	1	0.9	0.62	0.86
	Nitram	0.99	0.37	1	0.99	0.29	1	0.87	0.32	1.03
	SOP	0.99	1.97	1	0.99	1.72	1	0.88	2.13	1.24
Ni	Polysulphate	0.99	1.44	1.01	0.99	1.04	1	0.91	1.03	0.99
	DAP	0.97	0.93	1	0.99	0.53	1	0.94	0.44	0.88
	Nitram	0.52	0.51	1	0.99	0.21	1	0.85	0.21	1.02
	SOP	0.99	1.54	1.01	0.99	1.13	1	0.94	1.19	1.03
As	Polysulphate	0.99	1.47	1	0.99	1.44	1	0.9	1.42	0.89
	DAP	0.99	0.71	1	0.99	0.64	1	0.95	0.57	0.88
	Nitram	0.99	0.37	1.01	0.98	0.23	1	0.55	0.22	0.76
	SOP	0.99	1.68	1	0.99	1.46	1	0.99	1.64	1.13
Se	Polysulphate	0.99	1.76	1	0.97	1.58	1	0.8	1.62	1.02
	DAP	0.99	0.85	1	0.99	0.7	0.99	0.97	0.57	0.8
	Nitram	0.99	0.42	1.01	0.99	0.31	1.04	0.15	0.6	0.86
	SOP	0.99	1.93	1	0.99	1.78	0.99	0.92	1.8	1.01
Cd	Polysulphate	0.99	1.01	1.01	0.87	0.7	1	0.2	0.19	0.26
	DAP*	0.99	1.19	1	-	-	-	-	-	-
	Nitram*	0.98	0.83	1	-	-	-	-	-	-
	SOP	0.99	1.05	1.02	0.76	0.67	1.14	0.1	0.1	<0.1
Pb	Polysulphate	0.99	1.5	1	0.98	1.29	0.99	0.91	1.34	1.04
	DAP	0.99	0.37	1.01	0.99	0.68	0.99	0.96	0.59	0.88
	Nitram	0.99	0.37	1.01	0.99	0.27	1	0.6	0.24	0.85
	SOP	0.99	1.77	1	0.99	1.4	1	0.99	1.4	1

* < LOD.

Of the four fertiliser types (i.e. Polysulphate–sulphate; DAP–phosphate; Nitram–nitrate and SOP–potash) used for validation, the trace elements in Nitram, a straight N fertiliser made of ammonium nitrate were generally the worst predicted. This result could be attributed to the atomic weights of its components. As already stated, taking XRF measurements in air causes attenuation of low energy characteristic X-ray lines from low Z elements, thus less secondary fluorescent energy was detected to be quantified. An implication of this result is that, measurements must be made in the presence of vacuum to improve sensitivity when analysing nitrogen-based fertilisers primarily composed of elements with Z less than 11 and have low molecular weights such as urea, ammonium chloride and sodium nitrate. Another way around this limitation is to develop a calibration with only such fertilisers using current and voltage parameters that optimise the detection of low Z elements of interest. A low current and high voltage are good settings for low Z elements [33]. Thus, there is the indication that a calibration can be tailored and fine-tuned to a fertiliser type with target element(s) of interest for even better performance.

Using the two developed in-house models, the hand-held Tracer 5i determined the elemental composition of NIST SRM 695, a multi-nutrient blended fertiliser as given in Table 9. Several major (P, Ca), micro (Mn, Fe, Cu) and trace (Cr, Ni, As) elements are in the green zone. With their RSD values below 10, and % D values within the acceptable range of +/- 20% of the reference value, the results show that pXRF can give precise and accurate measurements of these elements in fertilisers [35].

**Table 9:** Elemental composition of NIST SRM 695.

Element	Reference values		pXRF values*					
	Ave	SD	Ave	SD	Min	Max	RSD	% D
P (%)	7.2	0.1	6.6	0.4	5.9	7.2	6.3	8.1
Ca (%)	2.3	0	1.8	0.1	1.7	2	4.9	18.5
Cr (mg kg^-1^)	244	6	257.3	14.5	227	288	5.6	5.5
Mn (%)	0.3	0	0.3	0	0.3	0.3	5.5	1.2
Fe (%)	4	0.1	3.4	0.2	3	3.7	5.4	13.8
Ni (mg kg^-1^)	135	2	145.7	7.3	132	159	5	7.9
Cu (mg kg^-1^)	1225	9	1040.2	60.5	917	1144	5.8	15.1
As (mg kg^-1)^	200	5	190.5	13.3	164	215	7	4.8
S (%) ^¶^	4.9	0.1	3.6	0.3	3.1	4.1	9	26.9
K (%)	11.7	0.1	16.1	0.7	14.8	17.3	4.5	38
Co (mg kg^-1^)	65.3	2.4	44.9	10	23	65	22.2	31.3
Pb (mg kg^-1^)	273	17	163.8	12.7	138	187	7.8	40
Mg (%)	1.8	0.1	0.8	0.2	0.5	1.1	22.9	55.3
Al (%)	0.6	0	0.3	0	0.2	0.3	10.5	54.2
Ti (mg kg^-1^)	310		130.7	26.9	82	176	20.6	57.9
Zn (%)	0.3	0	0.5	0.1	0.4	0.6	9.8	59.2
Cd (mg kg^-1^)	16.9	0.2	28	12.4	13	56	44.2	65.7
Na (%)	0.4	0	<LOD					
Se (mg kg^-1^)	2.1	0.1	18.5	1	17	21	5.6	782.1
Mo (mg kg^-1^)	20	0.3	1229.6	108.7	989	1420	8.8	6048.1

*pXRF values from 61 scans collected on 6 days. Samples were scanned for 30, 60, 90 or 120 seconds. Samples were also scanned as loose or packed powders in pXRF cups.

^¶^Reference value for S was determined in-house using Aqua Regia digestion and ICP-OES.

**Fig 5:**
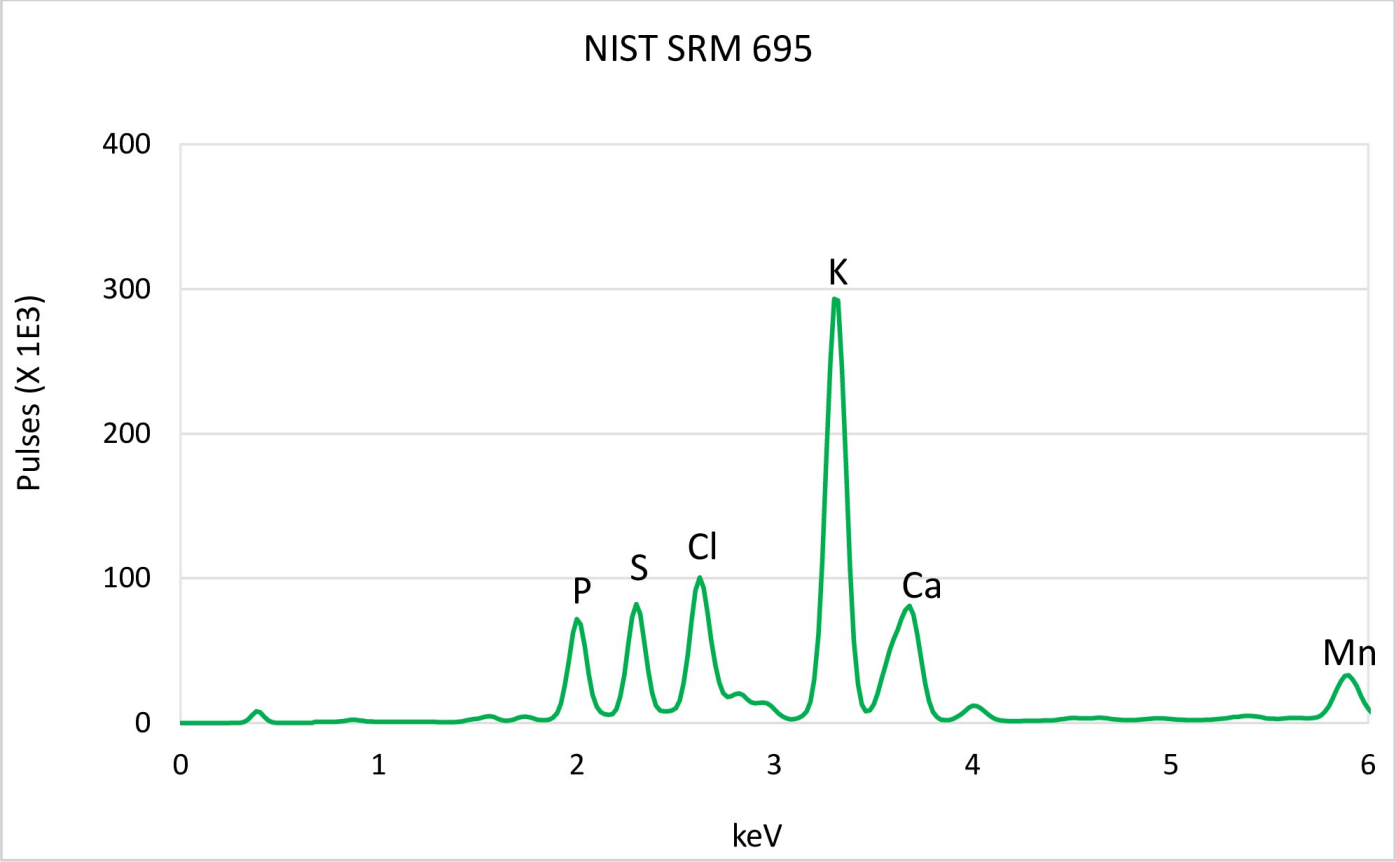
pXRF spectrum of NIST 695 showing energy lines for selected elements including for S.

No reference value was given for S in the certificate of analysis of NIST SRM 695. However, a mean S content of 3.6% was determined for this SRM using pXRF. Reviewing the spectra of this fertiliser showed the presence of S, Fig 5. As such, Aqua Regia digestion coupled with ICP-OES was employed in-house to ascertain this. The results confirmed the presence of S at a concentration of 4.9%. Thus, this study adds valuable information from a Wageningen Evaluating Programs for Analytical Laboratories (WEPAL) participating laboratory on S, a major nutrient to NIST SRM 695.

For the other elements of interest, pXRF did not work as well as it did for the 8 elements in the green zone. Nonetheless, the pXRF gave adequately precise measurements, with RSD values generally below the specified 20% [35], even for Se and Mo, the two elements that pXRF measured with the least accuracy in this study. Cd had the largest RSD value of 44.2%, a reflection of how the models developed in this study were not stable and robust enough at concentrations less than 100 mg kg^-1^. This was for instance shown in the analysis of NIST SRM 695 whereby no quantification value (i.e. <LOD) was sometimes given. The interferences of Rh and Pd mentioned earlier could be the cause of this. Finally, the Na content of NIST SRM 695 could not be determined with the current set of scanning parameters used on the pXRF.

The Instrumental Lower Level of Detection (ILLOD) could not be determined in this study due to the very limited number of standard reference materials for fertilisers. Future work could be done to develop internal standards for this exercise.

## Conclusions

Using fertilisers with a wide range of atomic weights and densities, this study had a goal to establish a robust global calibration that will capture potential future unknowns. Results obtained have demonstrated the potential of using pXRF for rapid characterization of fertilisers. Portable XRF can measure several major (P, Ca) and micro (Mn, Fe, Cu) nutrients, as well as trace elements and potential contaminants (Cr, Ni, As) in fertilisers with high accuracy and precision. The results obtained in this study is good, especially considering that loose powders were scanned for a maximum of 90 seconds without the use of a vacuum pump. However, for nitrogen-based fertilisers that are primarily composed of elements with Z less than or equal to 11, and have low molecular weights, measurements must be done in vacuum to improve sensitivity.

Portable XRF which has been traditionally utilized in the mining industry can be adopted by the fertiliser industry for quick screening by manufacturers, merchants or any other stakeholders concerned about the quality of raw materials and finished products, as well as institutions tasked to enforce quality standards in the industry and environment. In addition, when pXRF is equipped with a calibration, it can be used to rapidly ensure that the finished product is within specification, and environmental stewards can use it to check that potential trace element contaminants conform to limits.
